# Distinct Activity of Endocannabinoid-Hydrolyzing Enzymes MAGL and FAAH in Key Regions of Peripheral and Central Nervous System Implicated in Migraine

**DOI:** 10.3390/ijms22031204

**Published:** 2021-01-26

**Authors:** Adriana Della Pietra, Rashid Giniatullin, Juha R. Savinainen

**Affiliations:** 1 A. I. Virtanen Institute for Molecular Sciences, University of Eastern Finland, 70211 Kuopio, Finland; adriana.della.pietra@uef.fi; 2 Laboratory of Neurobiology, Kazan Federal University, 420008 Kazan, Russia; 3 Institute of Biomedicine, University of Eastern Finland, 70211 Kuopio, Finland

**Keywords:** migraine, pain, endocannabinoid, serine hydrolases, analgesia

## Abstract

In migraine pain, cannabis has a promising analgesic action, which, however, is associated with side psychotropic effects. To overcome these adverse effects of exogenous cannabinoids, we propose migraine pain relief via activation of the endogenous cannabinoid system (ECS) by inhibiting enzymes degrading endocannabinoids. To provide a functional platform for such purpose in the peripheral and central parts of the rat nociceptive system relevant to migraine, we measured by activity-based protein profiling (ABPP) the activity of the main endocannabinoid-hydrolases, monoacylglycerol lipase (MAGL) and fatty acid amide hydrolase (FAAH). We found that in trigeminal ganglia, the MAGL activity was nine-fold higher than that of FAAH. MAGL activity exceeded FAAH activity also in DRG, spinal cord and brainstem. However, activities of MAGL and FAAH were comparably high in the cerebellum and cerebral cortex implicated in migraine aura. MAGL and FAAH activities were identified and blocked by the selective and potent inhibitors JJKK-048/KML29 and JZP327A, respectively. The high MAGL activity in trigeminal ganglia implicated in the generation of nociceptive signals suggests this part of ECS as a priority target for blocking peripheral mechanisms of migraine pain. In the CNS, both MAGL and FAAH represent potential targets for attenuation of migraine-related enhanced cortical excitability and pain transmission.

## 1. Introduction

Migraine is a widespread neurovascular disabling disorder affecting up to 15% of the worldwide population and is typically characterized by one-sided throbbing long-lasting moderate or severe pain [1,2]. Migraine is associated with multiple psychiatric comorbidities such as anxiety, depression and panic disorders [3,4,5,6]. Despite this clear association, the CNS neuronal centers underlying the link between migraine and the comorbid psychiatric conditions remains to be determined. Migraine has a clear trend to chronicization, namely, a progression from episodic to chronic migraine [7]. This trend might be due to excessive use of analgesic including opioids drugs leading to condition known as a ’medication overuse headache’ [8]. Although cannabis can help in opioid detoxification [9], its abuse can trigger psychiatric risk factors for migraine such as anxiety and depression, which are common among cannabis users [10] and associated with the chronicization of migraine [4,11]. Also, the post-traumatic stress disorder has been shown to be associated with migraine and drug abuse [12]. The main complaint of migraine patients is long-lasting pulsating pain, which is intractable in many cases thus leading to chronic stress and depression [3,13,14]. Known from ancient times [15], cannabinoids emerged recently as a promising analgesic approach to treat migraine pain [16,17,18]. In particular, cannabis, now legalized in many countries, has shown a therapeutic effect in migraine [16,19]. Indeed, marijuana had been used in the past for medicinal purposes to relieve headaches [20]. However, the exocannabinoids, exogenous compounds from cannabis and marijuana, which bind and activate cannabinoid receptors, have many adverse psychotropic and other unwanted effects [21]. Psychotropicity may be as detrimental as the migraine condition itself for conducting everyday life. Therefore, an alternative approach for migraine pain therapy might be based on the selective enhancement of endogenous cannabinoids (endoCB), which are naturally generated in the nociceptive system in the body [22]. The Endocannabinoid System (ECS) is composed of endoCBs, a class of unique lipidic mediators including 2-arachidonoyl glycerol (2-AG) and anandamide (*N*-arachidonoyl ethanolamine, AEA), the metabolic enzymes for their synthesis and degradation along with the two G protein-coupled cannabinoid receptors (CB_1_ and CB_2_) [23]. The enhancement of endoCBs-activities is primarily important for conditions such as Clinical Endocannabinoid Deficiency (CECD), which was already proposed as a complication for several treatment-resistant types of pain, including migraine [16,24].

There are two major types of migraine, migraine with and without aura [25]. Whether the mechanisms initiating migraine attack are located in the CNS or in the periphery remains debated. The peripheral ‘trigeminovascular system’ (TGVS) is composed by meningeal nerves, vessels and immune cells. In migraine with aura, a plausible early event is the cortical spreading depolarization (CSD), which leads to global depolarization of neurons and glial cells [26,27]. The depolarization slowly propagates along the cortical areas and, leading to activation of the TGVS [26,27]. In the absence of CSD, for instance, in episodic migraine without aura, the mechanisms triggering migraine are not still clearly established. However, it has been shown that the potential trigger for migraine attack is psychogenic stress, which can precipitate or worsen migraine [28,29]. Such stress can promote the release of corticotropin-releasing hormone [29], directly activating meningeal mast cells that are closely interacting with the dural nerves in the TGVS [30,31,32]. Alternatively, stress can provoke sleep disturbances, also known to trigger migraine episodes [33,34]. Among other key mechanisms initiating migraine attack is the release of the neuropeptides such as calcitonin gene-related peptide (CGRP) and pituitary adenylate cyclase-activating peptide (PACAP) [35,36,37]. In most migraine cases, there is an involvement of CNS centers, including the brainstem nuclei and hypothalamus [38].

Taken together, these data suggest that migraine pain may be regulated at multiple levels, suggesting that pro-nociceptive signaling before or during an attack may be inhibited by endogenous analgesic molecules in the peripheral nervous system (PNS) or within the CNS.

The analgesic anti-nociceptive potential of cannabinoid CB_1_ receptors is well established [39,40]. Moreover, it has been shown that AEA, one of the key endoCBs, inhibited trigeminal neurons in animal models of migraine [41,42]. However, the activity profile of endoCB-degrading enzymes, monoacylglycerol lipase (MAGL) and fatty acid amide hydrolase (FAAH), targeting 2-AG and AEA respectively, is poorly studied within the nociceptive system. Normally, MAGL and FAAH activity maintains low physiological levels of endoCBs. High local MAGL and FAAH activity in the PNS and CNS can keep the endogenous analgesic action of endoCBs at low levels, giving rise to CECD [16,24]. Given that migraine pain is different in the pathogenesis from somatic pain, it needs analgesic agents specifically targeting TGVS. In this regard, one intriguing issue is whether the profile of MAGL/FAAH activity is different in the trigeminal ganglia (TG) implicated in migraine and dorsal root ganglion (DRG) involved in the transmission of somatic and visceral pain.

Given the contributing to migraine severity role of psychogenic stress, an additional promising line of anti-migraine therapy could be the activation of the ECS by selected plant cannabinoids combined with partner terpenes reducing the level of stress or severity of comorbid mood disorders [43,44].

Therefore, by proposing MAGL and FAAH as main targets for an innovative multitarget (analgesic and antidepressant) treatment for migraine, we studied their activity in the rat PNS and CNS, in areas important for the generation and propagation of migraine-specific pain signals. The activity of these enzymes was evaluated by a versatile chemoproteomic method, activity-based protein profiling (ABPP), utilizing for validation of specificity the recently developed potent and specific MAGL and FAAH inhibitors [45,46].

## 2. Results

### 2.1. Peripheral and Central Activity of the Endocannabinoid-Hydrolyzing Enzymes MAGL and FAAH

#### 2.1.1. MAGL Activity Prevails at Peripheral Level: Trigeminal Ganglia and Dorsal Root Ganglia

**Figure 1:**
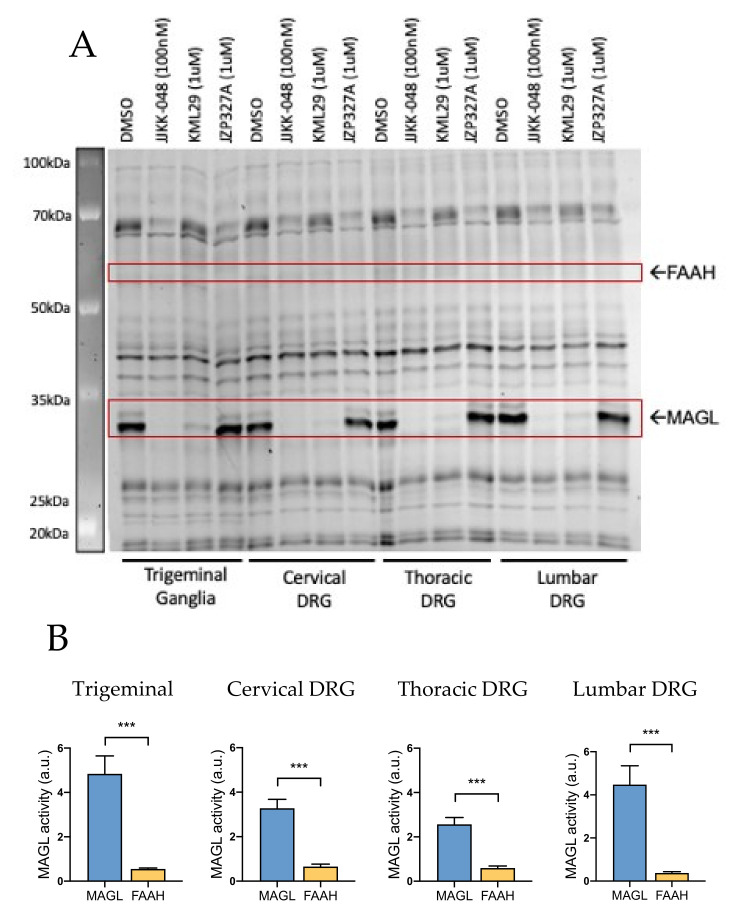
Competitive gel-based activity-based protein profiling (ABPP) reveals higher monoacylglycerol lipase (MAGL) activity over fatty acid amide hydrolase (FAAH) in trigeminal ganglia (TG) and cervical, thoracic and lumbar dorsal root ganglion (DRG). (**A**) Rat TG and DRG proteomes were preincubated for 1 h with vehicle (DMSO), the MAGL-inhibitors JJKK-048 (100 nM) and KML29 (1 μM) and FAAH-inhibitor JZP327A (1 μM). Then they were labeled with the fluorescent probe TAMRA-FP, as indicated in Materials and Methods. TAMRA-FP labeled bands (active serine hydrolases) appear dark after in-gel imaging. FAAH and MAGL were identified based on selective inhibition and their expected molecular weights. Notice that MAGL activity after DMSO treatment is high whereas the FAAH activity was almost absent. (**B**) Histograms comparing the basal activity of MAGL and FAAH in TG and DRG. Basal MAGL activity was approximately 9-fold higher compared that of FAAH in TG (in a.u., arbitrary units). Similarly, MAGL activity compared to that of FAAH was ~5-fold higher in cervical DRG, ~4-fold higher in thoracic DRG and ~11-fold higher in lumbar DRG. Unpaired t-test, *** *p* < 0.001, *n* = 8.

We found that both in TG and DRG, the basal MAGL activity (treatment with DMSO) was very high (Figure 1A). In both of these tissues, MAGL activity appeared as two MAGL-isoforms resulting a double-band. This activity was fully inhibited by the ultrapotent MAGL inhibitor JJKK-048 (100 nM) and almost totally blocked by the specific MAGL inhibitor KML29 (1 μM). A closer analysis of MAGL isoforms revealed that the short MAGL isoform appeared to be more active than the long isoform in peripheral tissues. However, both isoforms were equally active in the CNS (Supplementary Figure S1). Notice that, in contrast to MAGL, the basal FAAH activity (selectively inhibited by JZP327A) in rat TG and DRG was relatively low at these peripheral parts of the nociceptive system (Figure 1A).

Figure 1B shows that both rat TG and DRG have significantly higher MAGL activity compared to FAAH. This observation was particularly clear in TG, where the basal MAGL activity was approximately 9-fold higher compared to that of FAAH. Similarly, MAGL activity compared to FAAH was ~5-fold higher in cervical DRG, ~4-fold higher in thoracic DRG and ~11-fold higher in lumbar DRG.

#### 2.1.2. MAGL and FAAH Activity in Brainstem and Spinal Cord

**Figure 2:**
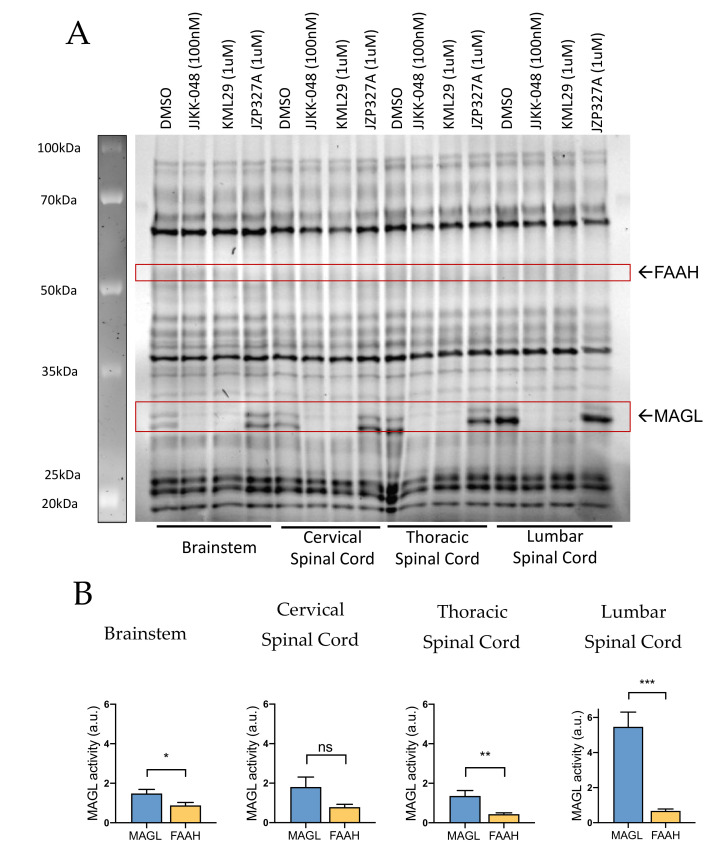
Competitive gel-based ABPP reveals variable MAGL and FAAH activities in rat brainstem and cervical, thoracic and lumbar spinal cord. (**A**) Brainstem and spinal cord proteomes were incubated for 1 h with vehicle (DMSO), MAGL inhibitors JJKK-048 (100 nM) and KML29 (1 μM) and FAAH inhibitor JZP327A (1 μM), and then labeled with the fluorescent probe TAMRA-FP, as indicated in Materials and Methods. FAAH and MAGL were identified based on selective inhibition and their expected molecular weights. Note that basal MAGL activity was high in the lumbar spinal cord but less intense in samples of the brainstem, cervical and thoracic spinal cord. Based on this analysis, FAAH activity was not found in samples of the brainstem and spinal cord. (**B**) Histograms comparing the basal activity of MAGL and FAAH in the brainstem and different spinal cord parts. Basal MAGL activity was approximately 2-fold higher compared to that of FAAH in brainstem, and ~4-fold higher in thoracic spinal cord. In the lumbar spinal cord, MAGL activity was ~8-fold higher than of FAAH. Unpaired *t*-test, * *p* < 0.05, ** *p* < 0.01, *** *p* < 0.001, ns = nonsignificant, *n* = 11 (BS, cSC) and *n* = 8 (tSC, lSC).

Next, to identify additional molecular targets for analgesia by affecting the most active ECS enzymes, we investigated the activity of MAGL and FAAH in central areas involved in the generation and transmission of migraine pain. ABPP testing of rat brainstem and cervical, thoracic and lumbar spinal cord samples revealed the presence of both MAGL and FAAH activity (Figure 2A). MAGL activity appeared high in the lumbar spinal cord, where it was ~8-fold higher compared to FAAH activity (Figure 2B). A lower relative basal MAGL activity against FAAH was observed also in the brainstem (~2-fold higher) and thoracic spinal cord (~4-fold higher). In contrast, no significant difference between basal MAGL and FAAH activities in the cervical spinal cord was found (Figure 2B).

#### 2.1.3. MAGL and FAAH Share the Spotlight at Central Cortical Level

**Figure 3:**
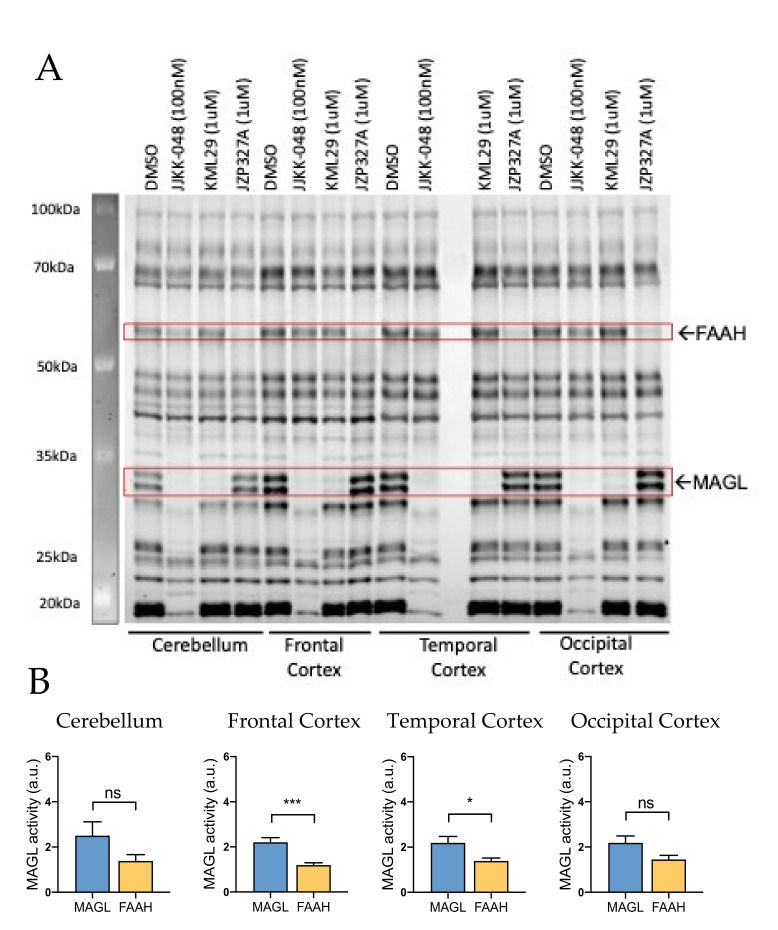
Competitive gel-based ABPP reveals MAGL and FAAH activity in rat cerebellum and cortex. (**A**) Cerebellar and frontal, temporal and occipital cortexes proteomes were incubated for 1 h with vehicle (DMSO), MAGL inhibitors JJKK-048 (100 nM) and KML29 (1 μM) and FAAH inhibitor JZP327A (1 μM), and then labeled with the fluorescent probe TAMRA-FP, as indicated in Materials and Methods. FAAH and MAGL were identified based on selective inhibition and their expected molecular weights. Both MAGL and FAAH activities were high in the cerebellum and cortex. (**B**) Histograms showing the basal activity of MAGL and FAAH in the cerebellum and frontal, temporal and occipital cortexes. Basal MAGL activity was ~2-fold higher compared to that of FAAH in frontal and temporal cortexes. In contrast, MAGL and FAAH activities were not found statistically different in samples of the cerebellum and occipital cortex. Unpaired t-test, * *p* < 0.05, *** *p* < 0.001, ns = nonsignificant, *n* = 10.

Next, we explored whether MAGL and FAAH were active also at the level of CNS. Using cerebellum and cortex samples (Figure 3A), we observed high MAGL and, for the first time, relatively high basal FAAH activity in cerebellum, frontal, temporal and occipital cortexes (Figure 3A). Indeed, in the cerebellum, frontal and temporal cortexes, the basal MAGL activity was only approximately 2-fold higher compared to that of FAAH (Figure 3B). No significant difference was observed between basal MAGL vs. FAAH activities in the occipital cortex, suggesting a comparable contribution of both of these hydrolases in control of endoCBs in this important for migraine area of the brain (Figure 3B).

### 2.2. MAGL has a Key Activity at Peripheral Level

**Figure 4:**
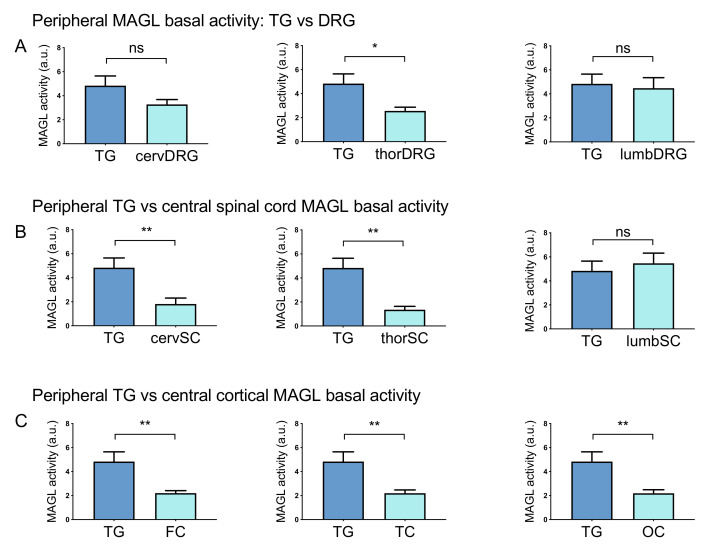
Comparing the basal MAGL activity in peripheral and central rat tissues. (**A**) Comparison of basal MAGL activity between TG and DRG. Basal MAGL activity was ~2-fold higher in TG than in thoracic DRG but no significant difference was found in TG vs cervical and lumbar DRG. (**B**) Comparison of basal MAGL activities between peripheral TG and CNS spinal cord tracts. Basal MAGL activity in the cervical (cervSC) and thoracic (thorSC) spinal cord was ~3-fold lower than in TG. The level of MAGL activity was similar between samples of TG and the lumbar spinal cord (lumbSC). (**C**) Comparison of basal MAGL activities between peripheral TG and cortical samples. Basal MAGL activity in frontal (FC), temporal (TC) and occipital cortexes (OC) was approximately half of that in the TG sample. Unpaired *t*-test, * *p* < 0.05, ** *p* < 0.01, ns = nonsignificant, *n* = 8 (TGs, DRGs), *n* = 11 (BS, cSC), *n* = 8 (tSC, lSC) and *n* = 10 (Cbl, cortex).

Given the overall prevalence of MAGL over FAAH in basal activity in most of the peripheral and central areas, we also investigated whether MAGL has a similar prevailing activity in certain areas of the PNS and CNS in comparison with a key migraine-related tissue such as TG. We found that MAGL activity was higher in peripheral TG than in most of the other areas (Figure 1A, Figure 2A, Figure 3A and Supplementary Figures S2–S4). In particular, Figure 4A shows that MAGL activity in TG was ~2-fold higher than in thoracic DRG. However, we could not find a significant difference among activities in TG and cervical and lumbar DRG (Figure 4A, Figure 1A). Basal MAGL activity in TG prevails on most of the other tissues: ~3-fold higher than in cervical and thoracic spinal cord and ~2-fold higher than in cortexes (Figure 4B,C, Figure 1A, Figure 2A, Figure 3A).

### 2.3. Inhibition of MAGL and FAAH in Peripheral and Central Nervous Tissues

#### 2.3.1. JJKK-048, KML29 and AKU-005 Block Basal MAGL Activity in Both Peripheral and Central Samples

**Figure 5:**
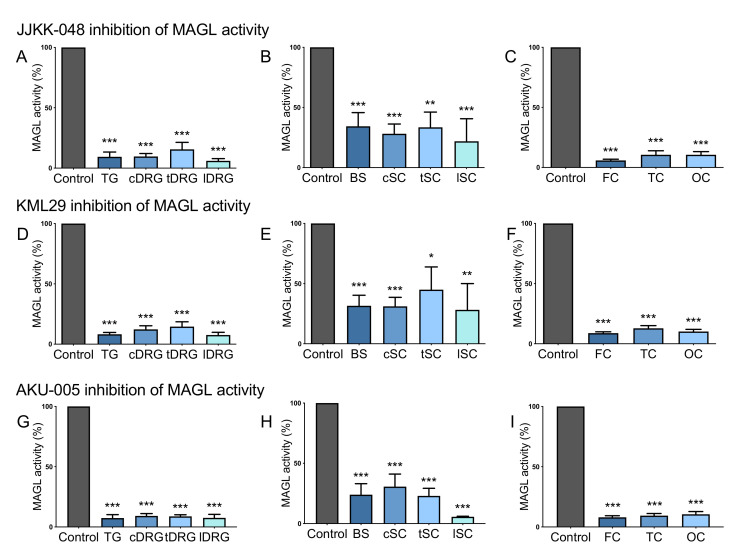
The blocking capacity of the MAGL inhibitors JJKK-048, KML29 and the dual MAGL-FAAH inhibitor AKU-005 in the CNS and the PNS. (**A**) JJKK-048 (100 nM) almost completely inhibited MAGL activity at peripheral level: by 90% in TG and cervical DRG (cDRG), 85% in thoracic DRG (tDRG) and 95% in lumbar DRG (lDRG). (**B**) JJKK-048 (100 nM) inhibited MAGL activity by 66% in brainstem (BS), 72% in cervical spinal cord (cSC), 68% in thoracic spinal cord (tSC), 79% in lumbar spinal cord (lSC). (**C**) JJKK-048 (100 nM) inhibited MAGL activity by 95% in frontal cortex (FC) and 90% in temporal and occipital cortexes (TC and OC). (**D**) KML29 (1 μM) strongly reduced MAGL activity at peripheral level: 92% TG, 88% cDRG, 86% tDRG, 93% lDRG. (**E**) KML29 (1 μM) inhibitory effect on brainstem and cSC was 69%, in tSC 55% and 72% in lSC. (**F**) KML29 (1 μM) strongly reduced MAGL activity at cortical level: by 92% in FC, 88% in TC, 90% in OC. (**G**) AKU-005 (1 μM) reduced MAGL basal activity at peripheral level: 93% in TG, 91% in cDRG, 92% in tDRG and 93% in lDRG. (**H**) AKU-005 (1 μM) inhibited basal MAGL activity also in brainstem (77%) and spinal cord (70% cSC, 78% tSC, 95% lSC). (**I**) AKU-005 (1 μM) inhibitory effect on cortical MAGL activity was of 92% in FC, 91% in TC and 90% in OC. One-way ANOVA with Tukey’s multiple comparison post-hoc test was used for statistical analysis between the MAGL activities after control (DMSO) and inhibitor treatments, (* *p* < 0.05, ** *p* < 0.01, *** *p* < 0.001). For JJKK-048: *n* = 8 (TGs, DRGs), *n* = 11 (BS, cSC), *n* = 8 (tSC, lSC) and *n* = 9 (cortex); For KML29: *n* = 8 (TGs, DRGs), *n* = 11 (BS, cSC), *n* = 8 (tSC, lSC) and *n* = 9 (cortex); For AKU-005: *n* = 4 (TGs, DRGs, BS, tSC, lSC), *n* = 5 (cSC, cortex).

In order to find the most efficient ways to block MAGL and FAAH activities on TG, DRG, brainstem, spinal cord and cortex, we numerically evaluated the inhibitory action on these tissues of the recently proposed MAGL inhibitors JJKK-048 and KML29, FAAH inhibitor JZP327A and the dual MAGL-FAAH inhibitor AKU-005 (Figure 5, Supplementary Figures S2–S4). In this testing, we used the fully effective concentrations of the inhibitors based on our previous studies, determining the dose-responses of these compounds [24,25,26].

We found that the specific MAGL inhibitor JJKK-048 (100 nM) almost completely inhibited the activity of this ECS enzyme in peripheral parts of the nociceptive system (represented by TG and DRG). In particular, MAGL activity was reduced by 90% in TG and cervical DRG, by 85% in thoracic DRG and by 95% in lumbar DRG (Figure 5A). Moreover, this treatment also inhibited MAGL activity in the CNS. Figure 5B illustrates a reduction of MAGL activity by 66% in the brainstem (BS), 72% in the cervical spinal cord (cSC), by 68% in the thoracic spinal cord (tSC) and by 79% in the lumbar spinal cord (lSC). JJKK-048-mediated inhibition at the cortical level was even stronger, with a 95% reduction of MAGL activity in the frontal cortex (FC) and 90% in temporal and occipital cortexes (TC and OC, Figure 5C).

Another selective MAGL inhibitor KML29 (1 μM) also strongly reduced MAGL activity in the PNS, including TG and DRG (by 92% in TG, 88% in cDRG, 86% in tDRG and 93% in lDRG, Figure 5D). Likewise, it also strongly reduced MAGL activity in the cerebral cortex (by 92% in FC, 88% in TC and 90% in OC; Figure 5F). Moreover, we observed a noticeable KML29-dependent inhibitory effect on basal MAGL activity on the brainstem (69%), cervical (69%), thoracic (55%) and lumbar spinal cord (72%) (Figure 5E).

The dual MAGL-FAAH inhibitor AKU-005 (1 μM) exhibited the same inhibitory effect as JJKK-048 and KML29; a strong reduction of MAGL activity was observed at peripheral (by 93% in TG, 91% in cDRG, 92% in tDRG and 93% in lDRG, Figure 5G) and cortical level (by 92% in FC, 91% in TC and 90% in OC, Figure 5I). Although, AKU-005 had a moderate inhibitory effect on MAGL activity in the brainstem (77%) and spinal cord (70% cSC, 78% tSC, we observed a 95% reduction in lSC, Figure 5H).

#### 2.3.2. JZP327A Blocks FAAH Activity in the Cerebral Cortex

**Figure 6:**
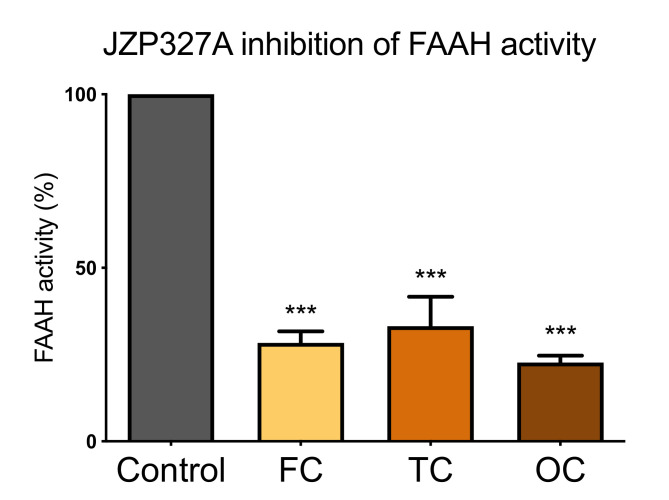
FAAH inhibitor JZP327A reduces basal FAAH activity in vitro at the cortical level. Data is from ABPP testing of the frontal cortex (FC), temporal cortex (TC) and occipital cortex (OC) proteomes incubated for 1 h with vehicle (DMSO) and the specific FAAH inhibitor JZP327A (1 μM). Notice that JZP327A reduced FAAH activity by 72% in FC, 67% in TC and 78% in OC. One-way ANOVA with Tukey’s multiple comparison post-hoc test was used for comparison, *** *p* < 0.001, *n* = 9.

Unlike the peripheral tissues, as well as the brainstem and spinal cord, we observed a significantly high basal FAAH activity only in cortical samples (Figure 3). This high activity, therefore, represented a reliable model to evaluate specific FAAH inhibitors. Indeed, the endoCB -hydrolyzing activity of FAAH in the cortex was readily blocked by the specific FAAH inhibitor JZP327A (1 μM) (Figure 6). Thus, JZP327A reduced basal FAAH activity by 72% in the frontal cortex (FC), 67% in the temporal cortex (TC) and 78% in occipital cortex (OC) samples.

## 3. Discussion

In this study, we evaluated, for the first time, the activity of the ECS metabolic enzymes in the PNS, including the peripheral trigeminovascular nociceptive system, and in the CNS areas such as the spinal cord, brainstem, cerebellum and cerebral cortex. These areas are involved in the generation and transmission of migraine pain as well as in other migraine-related events such as migraine aura. By utilizing a sensitive chemoproteomic ABPP assay, we profiled the activity of MAGL and FAAH, two major endocannabinoid-hydrolyzing enzymes in these tissues. Our data suggest MAGL as a potential peripheral neuronal target for the treatment of migraine pain. At the cortical level, where the activity of FAAH was similar to MAGL, the dual-inhibition of these enzymatic pathways can attenuate, via raising the levels of two main endoCBs 2-AG and AEA, the phenomenon of CSD, underlying aura and reduce the central pain transmission. We propose recently developed potent and selective MAGL and FAAH inhibitors for the activation of ECS in peripheral and central nervous structures involved in the anti-nociceptive signaling.

### 3.1. MAGL and FAAH Activity in Peripheral Nervous Systems

By utilizing the ABPP assay, we demonstrated the prevailing active state of the main endocannabinoid-degrading enzyme MAGL over FAAH in TG, which are the main constituent of the TGVS, the place where migraine pain originates from [36,47,48,49]. The activity of MAGL was higher than the respective activity of FAAH not only in TG, but also in DRG and brainstem, which are also implicated in the transmission of migraine pain [50,51]. Interestingly, we observed that the activity of MAGL was higher in TG than in some DRG suggesting the specific role of this pathway in trigeminal pain, including migraine headache.

One previous study reported high FAAH expression in rat DRG and spinal cord, suggesting a key role of AEA in modulating peripheral nociceptive signaling [52]. Notably, our data do not contradict this conclusion as the ABPP assay allowed us to estimate not only the expression level but also to detect the activity of serine hydrolases in the peripheral tissues that are implicated in the generation and transmission of migraine pain. The demonstration of the relatively high peripheral activity of MAGL over FAAH in neuronal tissues, suggests that any increase of 2-AG levels occurring at the periphery during a migraine attack would be largely damped down by high MAGL activity. This was a specific reason to suggest the treatment of migraine pain by the MAGL inhibitor to elevate the level of the anti-nociceptive 2-AG. However, within the TGVS, the generation of pain involves not only neurons but also vessels and immune cells, in particular, mast cells [32,53]. Thus, AEA could interfere with these immuno-vascular mechanisms in the meninges, ultimately leading to reduced nociception [54]. This can happen at the level of meningeal afferents or via the suppressed transmission of peripheral signals to the second-order brainstem neurons [16]. Moreover, the role of AEA could be enhanced during migraine states [55], which are known to be associated with intensive neuro-inflammatory processes [48]. Therefore, our data do not exclude the role of FAAH/AEA-signalling as a target for peripheral analgesia but suggests the MAGL/2-AG as the most straightforward target for anti-nociceptive treatments operating via neuronal mechanisms.

2-AG is the primary endoCB operating via inhibitory Gi/o-protein-coupled CB_1_ and CB_2_ receptors [16]. Indeed, the MAGL-substrate 2-AG was previously found to fully activate these receptors whereas the FAAH-substrate AEA behaves as a partial agonist at both receptors [23,56,57]. This view is consistent with our findings on the prevailing activity of MAGL over FAAH in neuronal tissues at the periphery.

Notably, apart from the accumulation of the analgesic endoCBs 2-AG and AEA, the inhibition of MAGL and FAAH has additional benefits for anti-nociception, by diminishing the level of endoCB degradation product arachidonic acid which is a precursor for the pro-inflammatory and pro-nociceptive prostaglandins [16]. Therefore, peripheral MAGL and FAAH inhibition may have a multicomponent effect on migraine and other types of inflammatory pain, mediated by mechanical hypersensitivity, and probably, neuropathic pain, which, like migraine, is characterized by allodynia.

### 3.2. MAGL and FAAH Activity in the Central Nervous Systems

In contrast to peripheral nociceptive pathways, the activity of FAAH was much higher in the CNS. However, the activity of MAGL remained high in the brain. The high activity of these two endoCB-degrading enzymes might indicate a relatively low tonic inhibitory role of both 2-AG and AEA in modulating central pain processes in healthy states. In the CNS, the level of 2-AG has some prevalence over AEA [57], suggesting 2-AG as the primary modulator of synaptic processes in the brain. Nevertheless, both 2-AG and AEA can serve as the common retrograde messengers released from post-synaptic membranes to target the inhibitory CB_1_ presynaptic receptors in glutamatergic and GABAergic synapses [23].

Together, these data indicate a high potential for pharmacological interventions in the ECS in order to activate, via endoCBs, the inhibitory CB_1/2_ receptors for the treatment of migraine pain.

The role of central neuronal networks and brain centers in migraine is region-specific. Thus, both the brainstem and cervical spinal cord (C1-C3) are implicated in the transmission of pain signals from the primary afferents to the second-order neurons [17,50,51]. Consistent with this, our study showed that in the cervical spinal cord, unlike other spinal cord areas, the activity of FAAH was not significantly different from MAGL, reflecting their specific role in migraine mechanisms.

The occipital cerebral cortex tested in our study, is the common area for the development of CSD, a phenomenon underlying aura in the specific form of migraine with aura [58]. Accordingly, CSD is likely giving rise to multiple visual abnormalities at the initiation of migraine attacks [59,60,61,62]. Temporal, frontal cortical lobes and the cerebellum were also reported to be altered in chronic migraine patients during pain signaling events [63,64,65,66]. In migraine-related cortex and cerebellum, we found the high activity of both MAGL and FAAH, suggesting a potential reserve for therapeutic interventions against the MAGL and FAAH activity by their specific inhibitors. Indeed, both MAGL and FAAH signaling have been shown to modulate pain transmission at central and peripheral levels [67]. It is generally accepted that CSD is an attractive target for anti-migraine agents [68]. Moreover, the suppression of CSD by activating CB_1_ receptors has already been shown [69], implying that similar effects may be achieved via activation of ECS.

Thus, the enhancement and anti-nociceptive signaling of endoCBs, 2-AG and AEA, via MAGL and FAAH inhibition, can provide a beneficial reduction of the excessive cortical excitability and attenuate the central pain transmission in migraine and in inflammatory or neuropathic pain.

### 3.3. Novel Endocannabinoid Hydrolase Inhibitors for the Treatment of Migraine

The identification of the ECS in several CNS areas presents an avenue to pharmacologically enhance the beneficial role of endoCBs in several pathological conditions, including pain, cancer, addictive behavior, epilepsy and psychiatric diseases [22,41]. In this study, we showed for the first time, the comprehensive profile of activity and specific inhibition of endoCB-hydrolyzing enzymes MAGL and FAAH in tissues of origin and transmission of migraine pain.

The majority of previously tested MAGL inhibitors lack high selectivity among different hydrolases [70]. In contrast, the recently found KML29 [46] compound has high MAGL-specificity and has been validated for its analgesic and anti-allodynic effects in vivo [71,72,73,74]. We propose the newly-synthesized highly potent MAGL inhibitor JJKK-048 (IC_50_ < 0.4 nM) [46,75] as strong prototype drug candidate for migraine analgesia.

Previous studies on FAAH inhibition using OL-135, URB597 [76,77] and PF3845 showed analgesic effects [72,76]. These results raised further interest in the application of FAAH inhibitors to different pain states and the identification of more efficient and selective compounds. In our study, we used the recently developed selective FAAH inhibitor JZP327A [45], which completely blocked FAAH activity in cerebral cortex samples.

An alternative and powerful tool for targeting both MAGL and FAAH in either TGVS and CNS is the recently developed dual MAGL-FAAH inhibitor AKU-005 that showed a strong inhibitory effect, even at nanomolar concentrations (IC_50_ value 0.2–1.1 nM) [46]. Moreover, another dual inhibitor JZL195 has been reported to elicit stronger pain relief than the other selective MAGL or FAAH inhibitors [78].

### 3.4. Summary

In summary, we observed distinct profiles of MAGL and FAAH activity in healthy PNS and CNS. We show that novel selective MAGL and FAAH inhibitors can fully block the peripheral and cortical activity of these endCB-degrading enzymes in vitro. Our findings highlight MAGL and FAAH as promising targets for novel anti-migraine strategies via selective enhancement of the anti-nociceptive endoCB drive in this common neurological disorder. Future research efforts may focus on testing novel MAGL and FAAH inhibitors in in vivo models of migraine.

## 4. Materials and Methods

### 4.1. Animals

Animal House of the University of Eastern Finland provided male Wistar rats for this study. For testing MAGL and FAAH activity, experiments were conducted on organ samples from 10–12 rats, on occipital cortex slices from 8 rats and trigeminal ganglia fragments from 7 rats. Animals were housed under the following conditions: 12h dark/light cycle, grouped housing, ad libitum access to food and water, at an ambient temperature of 22 °C. All experimental procedures performed in this study follow the rules of the European Community Council Directive of 22 September 2010 (2010/63/EEC). The Animal Care and Committee of the University of Eastern Finland approved all experimental protocols (licence EKS-008-2019, protocol from 25 November 2019).

### 4.2. Animals Dissection

4-6 weeks male Wistar rats were dissected according to published protocols to isolate the trigeminal ganglia [79], cortical areas, brainstem, cerebellum [80] dorsal root ganglia and spinal cord [81]. The spinal cord was divided into cervical (C2-C8), thoracic (T1–T13) and lumbar (L1–S4) tracts. We dissected cervical, thoracic and lumbar DRGs following the same vertebral segmentation.

### 4.3. Activity-Based Protein Profiling of Serine Hydrolases

Organ samples were mechanically homogenized (glass-glass homogenizer) in ice-cold PBS, and protein concentrations were determined with BCA protein assay (Pierce, Rockford, IL, USA), as previously described [82]. Competitive ABPP using tissue homogenates was conducted to visualize the selectivity of inhibitors toward endocannabinoid hydrolases FAAH and MAGL and against other serine hydrolases in tissue proteomes. We used the active site serine-targeting fluorescent fluorophosphonate probe TAMRA-FP (ActivX Fluorophosphonate Probes, Thermo Fisher Scientific Inc., Rockford, IL, USA) as previously described [83]. Briefly, tissue homogenates (100 μg protein) were pre-treated for 1 h with DMSO or the selected MAGL inhibitors JJKK-048 (School of Pharmacy, UEF) and KML29 (Cayman Chemicals (Ann Arbor, MI, USA), FAAH inhibitor JZP-327A (School of Pharmacy, UEF) or the dual MAGL/FAAH inhibitor AKU-005 (School of Pharmacy, UEF) with indicated concentrations, after which TAMRA-FP incubation was conducted for 1 h at room temperature (final probe concentration 2 μM) to label active serine hydrolases. The reaction was quenched by adding 2× gel loading buffer, after which 10 μg protein was loaded per lane and the proteins were resolved in 10 % SDS-PAGE together with molecular weight standards. TAMRA-FP labelled proteins were visualized by ChemiDoc™ MP imaging system (BIO-RAD, Hercules, CA, USA) with Cy3 blot application (602/50, Green Epi, Manual Exposure 10s–120s). Quantification of bands was performed by the software ImageLab (2020 Bio-Rad Laboratories) on the basis of band intensity (MAGL/FAAH activity, a.u.).

### 4.4. Statistical Analysis

Data were analyzed using GraphPad Prism 8 (GraphPad Prism Software, La Jolla, USA). The data are presented as mean ± SEM (standard error of the mean). Student’s unpaired *t*-test and One-way ANOVA with Tukey’s multiple comparison post-hoc test were used to detect statistical significances.

## Data Availability

The data that support the findings of this study are available from the corresponding author upon reasonable request.

## Supplementary Materials

Supplementary materials are available online at https://www.mdpi.com/1422-0067/22/3/1204/s1, can be found in the file “Supplementary materials—Molecular keys for migraine treatment via selective inhibition of endocannabinoid-hydrolyzing enzymes”.

## Abbreviations

2-AG	2-arachidonoyl glycerol
ABPP	Activity-based protein profiling
AEA	N-arachidonoyl ethanol amine, anandamide
BS	Brainstem
CB_1_, CB_2_	Cannabinoid receptors 1, 2
Cbl	Cerebellum
cDRG	Cervical dorsal root ganglia
CECD	Clinical endocannabinoid deficiency
cerv	Cervical
CGRP	Calcitonin generelated peptide
CNS	Central nervous system
cSC	Cervical dorsal root ganglia
CSD	Cortical spreading depression
DMSO	Dimethyl sulfoxide
DRG	Dorsal root ganglia
ECS	Endocannabinoid system
endoCB	Endocannabinoids
FAAH	Fatty acid amide hydrolase
FC	Frontal cortex
lDRG	Lumbar dorsal root ganglia
lSC	Lumbar spinal cord
lumb	Lumbar
MAGL	Monoacylglycerol lipase
NMDA	N-methyl-D-aspartate
NMDAR	N-methyl-D-aspartate receptors
OC	Occipital cortex
PACAP	Pituitary adenylate cyclase-activating enzyme
PNS	Peripheral nervous system
TC	Temporal cortex
TG	Trigeminal ganglia
TGVS	Trigeminovascular system
tDRG	Thoracic dorsal root ganglia
thor	Thoracic
tSC	Thoracic spinal cord
