# Supplementary material for: Distinct Activity of Endocannabinoid-Hydrolyzing Enzymes MAGL and FAAH in Key Regions of Peripheral and Central Nervous System Implicated in Migraine
Source: Int J Mol Sci. 2021 Jan 26;22(3):1204. doi: 10.3390/ijms22031204 (PMC7865507; doi:10.3390/ijms22031204)
Supplement: Supplementary file 1 [file ijms-22-01204-s001.pdf]

# Molecular keys for migraine treatment via selective inhibition of endocannabinoid-hydrolyzing enzymes

Adriana Della Pietra, Rashid Giniatullin and Juha R. Savinainen

## Supplementary materials

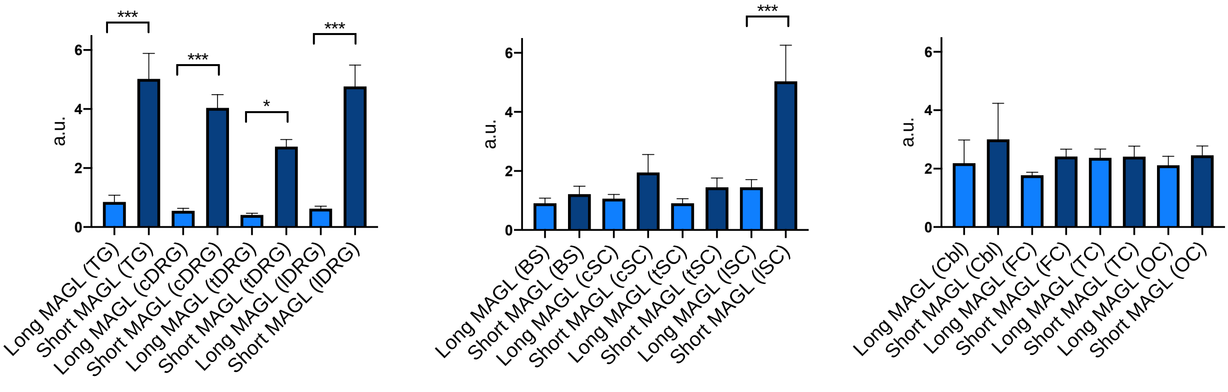


**Figure S1. Comparison between short and long MAGL isoform activity in PNS and CNS. (A)** Comparison of the basal activity of short versus long MAGL in TG and DRG. Activity of short MAGL activity is approximately 6-fold higher compared to that of long isoform in rat TG. Similarly, short MAGL activity compared to long isoform activity is ~8-fold higher in cervical DRG, ~7-fold higher in thoracic DRG and ~8-fold higher in lumbar DRG (one-way ANOVA with Tukey multiple comparison post-hoc test, ***$p<0.001$, *$p<0.05$, n=8). **(B)** Comparison of the basal activity of short and long MAGL isoforms in brainstem (BS) and spinal cord (SC). Activity of short MAGL is approximately ~4-fold higher in lumbar SC. There is no significant difference between activities of short and long MAGL isoforms in BS, cervical and thoracic SC (one-way ANOVA with Tukey multiple comparison post-hoc test, ***$p<0.001$, n=8). (**C**) Comparison of the basal activity of short and long MAGL isoforms in cerebellum (Cbl) and cortex. There are no significant differences between the activity short and long MAGL isoforms in Cbl and cortex (one-way ANOVA with Tukey multiple comparison post-hoc test, n=8).

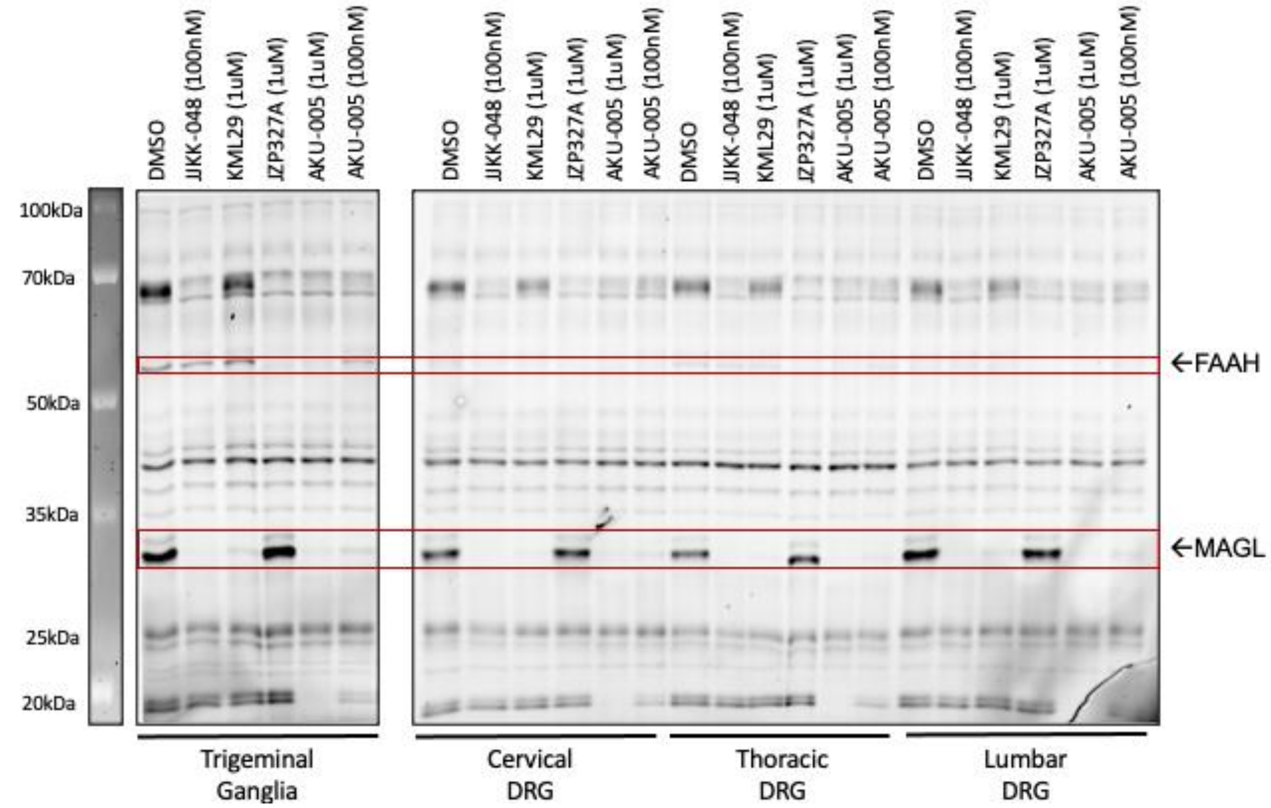


**Figure S2. Competitive ABPP reveals MAGL and FAAH inhibition in TG and DRG.** Rat TG and DRG proteomes were first preincubated for 1 h with vehicle (DMSO), the MAGL-inhibitors JJKK-048 (100 nM) and KML29 (1 μM), FAAH-inhibitor JZP327A (1 μM) and dual MAGL-FAAH inhibitor AKU-005 (100 nM and 1 μM). Then, they were labelled with the fluorescent probe TAMRA-FP, as indicated in Materials and Methods. FAAH and MAGL were identified based on selective inhibition and their expected molecular weights (marker lines on the left from stain free picture of the same gel). MAGL and FAAH band-intensities after DMSO treatments represent basal MAGL and FAAH activities, respectively. Note that JJKK-048 (100 nM) and KML29 (1 μM) selectively inhibited the basal MAGL activity in both TG and DRG. JZP327A-(1 μM) induced inhibition of FAAH is evident in TG samples but almost negligible in DRG due to very low FAAH activity. The dual MAGL-FAAH inhibitor AKU-005 (tested at 1 μM and 100 nM) fully blocked MAGL-activity at 1 μM, but has off-targets at this concentration (low molecular weight serine hydrolases) whereas at lower concentration (100 nM), it was selective for MAGL. Note that the inhibition of FAAH by AKU-005 (1 μM and 100 nM) is not visible due to low basal FAAH activity in DRG.

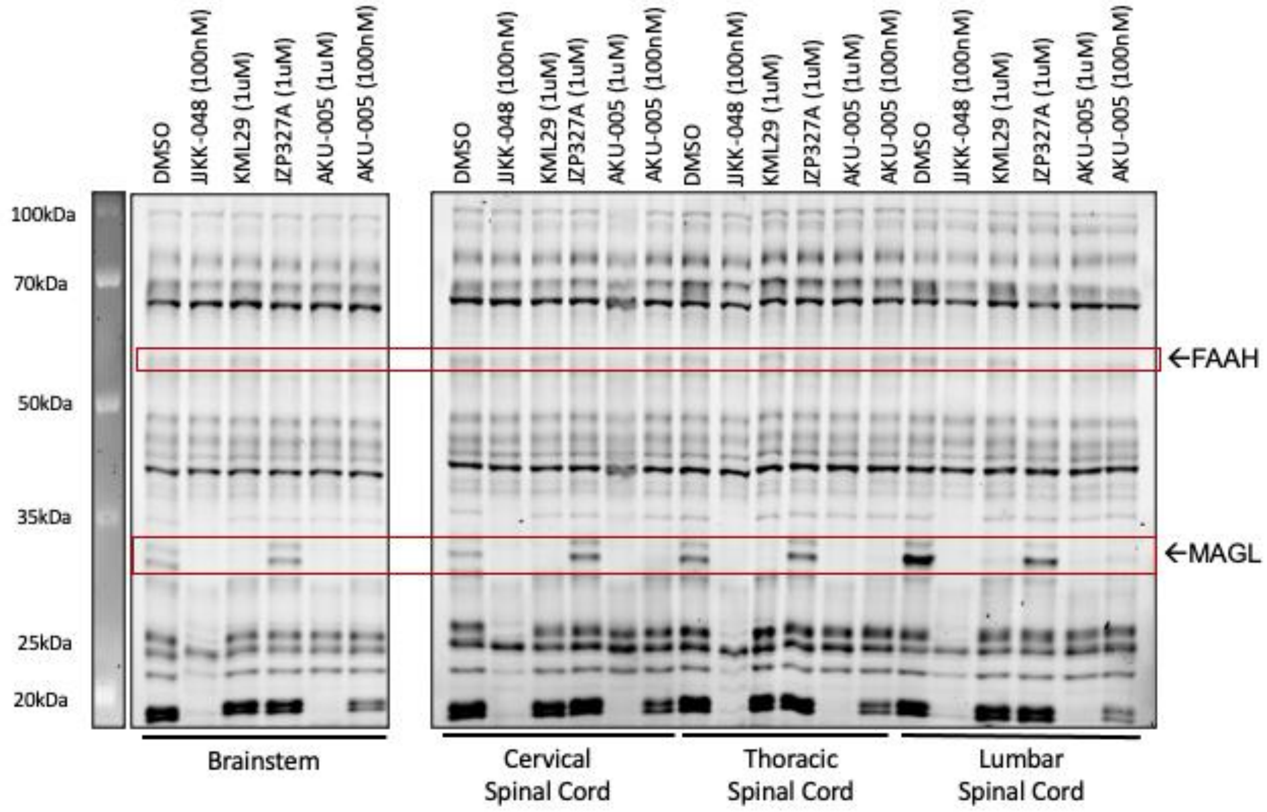


**Figure S3. Competitive ABPP reveals MAGL and FAAH inhibition in brainstem and spinal cord.** Rat brainstem and spinal cord proteomes were preincubated for 1 h with DMSO (vehicle), MAGL-inhibitors JJKK-048 (100 nM) and KML29 (1 μM), FAAH-inhibitor JZP327A (1μM) and dual MAGL-FAAH inhibitor AKU-005 (100 nM and 1 μM ). Then, they were labelled with the fluorescent probe TAMRA-FP, as indicated in Materials and Methods. FAAH and MAGL were identified based on the selective inhibition and their expected molecular weights (marker lines on the left from stain free picture of the same gel). MAGL and FAAH band-intensities after DMSO treatments represent basal MAGL and FAAH activities, respectively. Note that JJKK-048 (100 nM) and KML29 (1 μM) selectively inhibited the basal MAGL activity in both brainstem and spinal cord samples. FAAH activity was relatively low in these samples and activity can be inhibited by JZP327A (1 μM) in both TG and DRG. The dual MAGL-FAAH inhibitor AKU-005 (tested at 100 nM and 1 μM) fully blocked MAGL and FAAH activity but at 1 μM concentration, it has additional low molecular weight off-targets other than MAGL and FAAH.

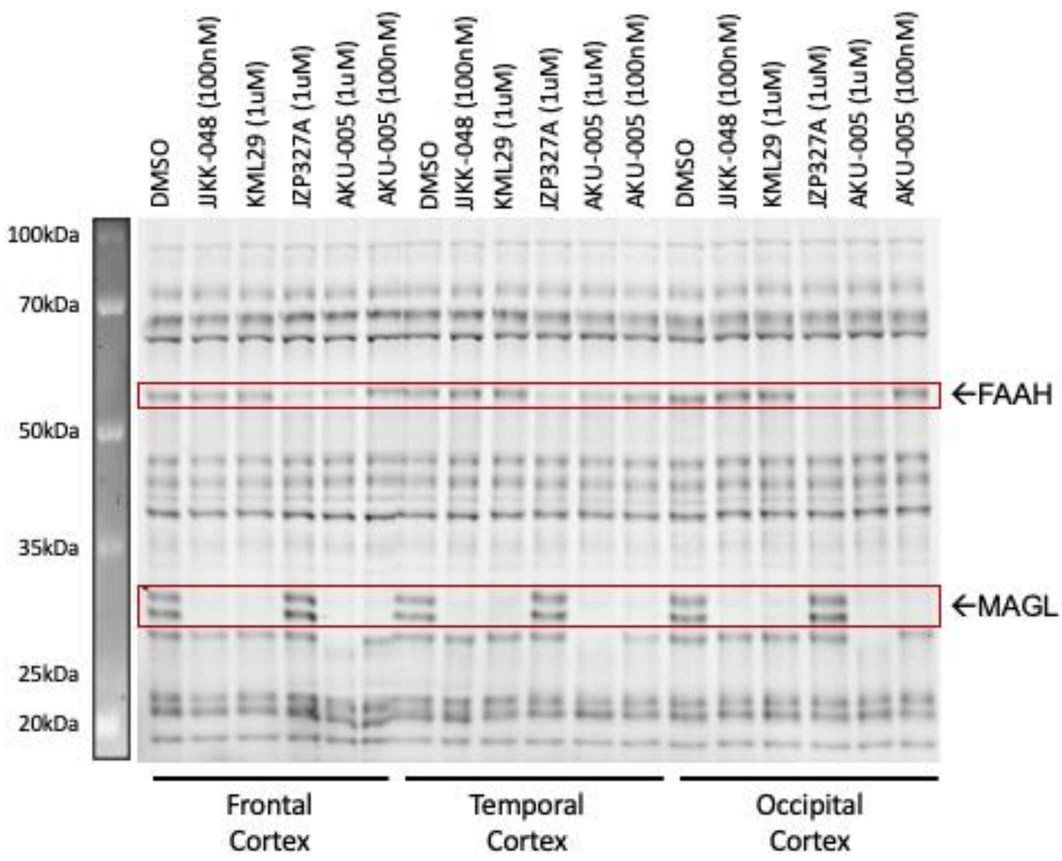


**Figure S4. Competitive ABPP reveals inhibition of MAGL and FAAH in cortical areas.** Rat cortical proteomes were preincubated for 1 h with vehicle (DMSO), MAGL-inhibitors JJKK-048 (100 nM) and KML29 (1 μM), FAAH-inhibitor JZP327A (1 μM) and dual MAGL-FAAH inhibitor AKU-005 (100 nM and 1 μM). Then, they were labelled with the fluorescent probe TAMRA-FP, as indicated in Materials and Methods. FAAH and MAGL were identified based on selective inhibition and their expected molecular weights (marker lines on the left from stain free picture of the same gel). MAGL and FAAH band intensities after DMSO treatments represent the basal MAGL and FAAH activities, respectively. Note that JJKK-048 (100 nM) and KML29 (1 μM) selectively inhibited the basal MAGL activity in frontal, temporal and occipital cortexes. JZP327A (1 μM) inhibited FAAH- activity in all cortical areas. The dual MAGL-FAAH inhibitor AKU-005 (100 nM and 1 μM) blocked MAGL and FAAH activity at 1 μM but was not sufficiently effective for FAAH inhibition at 100 nM concentration.
